# Universal electronic-cigarette test: physiochemical characterization of reference e-liquid

**DOI:** 10.1186/s12971-017-0119-x

**Published:** 2017-02-16

**Authors:** Jeffrey J. Kim, Nicole Sabatelli, Wojtek Tutak, Anthony Giuseppetti, Stanislav Frukhtbeyn, Ian Shaffer, Joshua Wilhide, Denis Routkevitch, John M. Ondov

**Affiliations:** 1 Clinical Research, Volpe Research Center, ADA Foundation, 100 Bureau Drive Stop 8546 NIST, Gaithersburg, MD 20899 USA; 2 School of Engineering, University of Maryland College Park, College Park, MD USA; 3 Molecular Characterization and Analysis Complex, University of Maryland Baltimore County, Baltimore, MD USA; 4 Department of Biomedical Engineering, Johns Hopkins University, Baltimore, MD USA; 5 Department of Chemistry and Biochemistry, University of Maryland College Park, College Park, MD USA; 6 Present address: Food and Drug Administration, Silver Spring, MD USA

**Keywords:** Electronic-cigarette, e-cig, Reference material, e-liquid, Universal testing method

## Abstract

**Background:**

Despite the rising health and safety concerns of e-cigarettes, a universal e-cigarette testing method is still in its early developmental stage. The aim of this study was to develop an e-liquid Reference Material that can be used to improve accuracy and reproducibility of research results, and advance health risk assessment of e-cigarette products.

**Methods:**

E-liquid Reference Material was developed by purity assessment, gravimetric measurement, homogeneity testing, and stability testing with material and instrument traceability (adopted from ISO 35:2006E).

**Results:**

Homogeneity tests showed e-liquid Reference Material requires ≥ 1 h rotation at a speed of 5 rpm to reach complete homogeneity. Stability tests showed homogeneity is intact for at least 2 weeks without secondary separation, and e-liquids are stable in 21 °C–50 °C thermocycling conditions up to 72 h. A change in the e-liquid color was first observed at day seven, and progressed to 2- and 16 - fold increase in absorbance by one and 6 months respectively. We found that e-liquids do not have inherent material instabilities such as immiscibility or secondary separation. However, discrepancies in concentration and composition arose mainly due to viscosity of propylene glycol and glycerin. Aerosol generated from the e-liquid Reference Material had 16 chemical-byproducts and was composed of ~634,000 particles of which 38% were Fine Particulate Matters (<0.5 μm in diameter).

**Conclusions:**

The efforts described here to create a standardized e-liquid Reference Material aim to provide unbiased and robust testing parameters that may be useful for researchers, the industry and government agencies. Additionally, the reference e-liquid could open a channel of conversation among different laboratories by providing the means of independent verification and validation while establishing a system of transparency and reproducibility in materials and methods.

## Background

The electronic cigarette industry has been doubling its annual growth, overtaking combustible cigarette sales among U.S. young adults in 2014; its global projected sales will reach $10 billion by 2017 [1, 2]. The latest studies show 79% of U.S. consumers recognize e-cigarettes and 44% believe e-cigarettes are less harmful than traditional cigarettes despite warnings from the World Health Organization (WHO), U.S. Food and Drug Administration (FDA), Centers for Disease Control and Prevention (CDC), American Medical Association, American Lung Association, and American Dental Association [3–9]. In 2014, 12.6% of U.S. and 11.6% of European adults have tried e-cigarettes at least once [10, 11]. France had the highest number of adult e-cigarette users (21.3%), while Portugal had the lowest (5.7%) [11]. There are approximately 9 and 2.1 million regular e-cigarette users in the U.S. and the U.K., respectively [10, 12]. The success of the e-cigarette industry, in part, can be attributed to aggressive marketing that targets specific age groups and the public’s perception that e-cigarettes are a safer alternative to traditional tobacco products [13, 14].

In the U.S., over 400 companies distribute thousands of products through local “vape shops” and online stores with little or no regulation [15]. Most products are imported from China which is the largest manufacturer of e-cigarettes, producing 95% of the world’s e-cigarettes, primarily for the European and the U.S. consumer markets [16]. Most companies do not disclose ingredients in e-liquids or provide proof of safety, good manufacturing practices and/or quality control measures [17–19]. Several studies have reported significant differences between nicotine concentrations indicated by manufacturers vs. the actual concentrations verified by independent laboratories [20–26]. More concerning is the detection of various levels of nicotine in “nicotine-free” e-liquids [21, 22, 26]. In addition, unapproved pharmaceutical ingredients - metals, carcinogens, toxic chemicals, and industrial grade propylene glycol - have been identified in commercially available e-liquids [27, 28]. In 2014, the Australia Department of Health implemented strict regulations under the Liquid Nicotine and Personal Importation for Use in E-cigarettes Guideline [29]. In 2016, European Union Member States started to regulate e-cigarettes as part of the EU Tobacco Products Directive [30]. In 2016, the U.S. Food and Drug Administration amended the 2009 Family Smoking Prevention and Tobacco Control Act by extending its authority over e-cigarettes, cigars and all other tobacco products [31]. The effectiveness of policy implementation on public health and its impact on the e-cigarette industry remain to be seen.

Many contributing factors to inconsistent and contradicting findings in e-cigarette research include lack of standardized research materials, testing devices and test methods. These inadequacies remain a major hurdle in bringing clarity to the situation [19, 25, 32]. Considering there are over a thousand e-liquid formulations, many with significant quality variations, it is not feasible to test every product. This challenge is not new to the manufacturing industry. Manufacturers have applied various quality control practices to improve the manufacturing process and product quality. One of the most common methods is implementing a Reference Material (RM) in the quality control process. RM is a matrix-matched material with assigned target values and assigned ranges for each variable, reliably determined from data obtained by repeated analysis [33]. RMs are routinely used to check the quality and metrological traceability of products, as well as for instrument calibration. The Reference Cigarettes produced by the Center for Tobacco Reference Products (University of Kentucky), for example, have provided much needed standards for tobacco manufacturers, government agencies and research institutions. Similar reference products have not been available in the e-cigarette research field until now. Recently, the British Standards Institution (BSI) and Association Française de Normalisation (AFNOR), with support from tobacco product manufacturers and the electronic cigarette industry trade association, published PAS 54115:2015 and XP D 90–300–2 respectively. Both technical specifications explain aspects of manufacturing standards and analytical testing methods in detail mainly from the industry perspective. However, development and utility of e-liquid RM was not explicitly discussed in these specifications.

In this study, we describe development of an e-liquid RM and physiochemical characterization of aerosol in support of establishing universal e-cigarette testing parameters. The e-liquid RM can serve as a key component in the proper experimental design process to improve accuracy, transparency, and reproducibility of data. For example, current e-cigarette studies use combustible cigarettes, Nicorette gums, nicotine inhalers and/or lozenges as a control [1]. Although their use is well justified for comparative analyses (e.g. the amount of harmful and potentially harmful constituents (HPHCs) in e-cigarettes vs. combustible cigarettes), e-liquid RM is necessary for two reasons: (1) the chemical composition and delivery process are unique in e-cigarettes compared to other nicotine and tobacco products which makes direct comparison between the two difficult, and (2) the quality of e-liquids and e-cigarette devices vary so widely that researching with less-reliable commercial products will inevitably lead to inconsistent final outcomes. The e-liquid RM will allow users to check the quality of unknown e-liquids, perform instrument calibration, assess toxicological risks, and test safety and efficacy of e-cigarette devices.

Currently, there is considerable variance in e-liquids and in testing devices. With so many variables it is difficult to perform meaningful studies that permit comparison of results among independent laboratories. This study aims to develop an e-liquid RM that can be used to compare different e-cigarette devices and e-liquids, assess various testing parameters, and improve reproducibility in e-cigarette research.

## Methods

### E-liquid Reference Material (RM)

#### Starting materials with specified properties

**Fig. 1:**
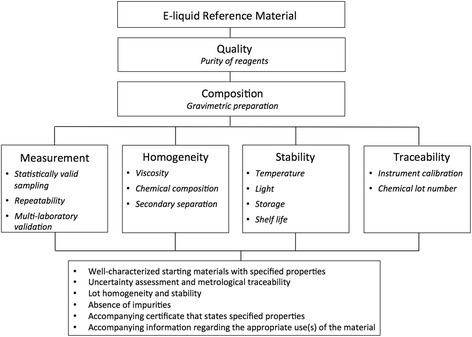
A Standard Operating Procedure (SOP) for e-liquid Reference Material formulation by adopting ISO Guide 35:2006E (Reference Materials: General and Statistical Principles for Certification) guideline. The reference e-liquid was characterized by measuring quality, homogeneity, stability and traceability of each component of the e-liquid

E-liquid RM was prepared by combining propylene glycol, glycerin and nicotine. Propylene glycol (Sigma Aldrich 49770) and glycerin (Sigma Aldrich 82280) were puriss. p.a., analytical grade chemicals that met American Chemical Society (ACS) specifications. They were tested to be >99.9% pure by Gas Chromatography (GC) and can be traced by Lot Numbers SZBE279CV and BCBN5225V, respectively. Nicotine solution (Sigma Aldrich N3876) was tested to be 99.5% pure (Lot Number 1449194 V) (Fig. 1).

#### Gravimetric preparation

An analytical micro-scale (A&D) was used to weigh 1.036 g propylene glycol, 1.250 g glycerin and 20.0 mg nicotine to make the reference e-liquid (1:1 propylene glycol/glycerin (v/v) with 10 mg/ml nicotine) as shown in Fig. 1. 8:2 and 2:8 propylene glycol/glycerin e-liquid RMs were prepared by weighting 1.650 g propylene glycol, 0.500 g glycerin and 20.0 mg nicotine; and weighting 0.410 g propylene glycol, 2.000 g glycerin and 20.0 mg nicotine, respectively.

#### Homogeneity tests

Within-bottle homogeneity (based on the ISO 35:2006E guideline) was measured by rotating 1 mL e-liquid RM in a 7.5 mL round-bottom test tube (13 × 100 mm, Pyrex) using a laboratory rotator (230401 V, VWR) set at 5 revolutions per min (r.p.m.) (Fig. 1). At 5 min, 20 min, 1 h, 3 h and 6 h timepoints, three subsamples were collected from the e-liquid RM and saved for nicotine concentration measurement (Table 1). Next, secondary separation was measured by leaving a homogenous 1:1 propylene glycol/glycerin e-liquid RM undisturbed for 24 h and 2 weeks (Table 1).

**Table 1:** E-liquid RM homogeneity and stability tests^a^

Variable	Sub-sample 1	Sub-sample 2	Sub-sample 3	*S.D.*	# of liquid phase
Nicotine concentration (mg/ml)					
Homogeneity test (1:1)					
5 min	10.65	9.19	8.93	0.92	2
20 min	9.90	9.79	9.55	0.17	2
1 h	9.69	10.00	9.78	0.15	1
Homogeneity test (8:2)					
5 min	10.41	10.29	10.23	0.31	2
20 min	10.48	10.53	11.82	0.66	2
1 h	10.89	10.61	11.00	0.20	1
Homogeneity test (2:8)					
5 min	9.28	8.49	7.98	0.59	2
20 min	9.63	8.44	9.63	0.64	2
1 h	9.73	9.36	8.33	0.73	1
3 h	9.54	8.91	9.14	0.32	1
6 h	10.99	9.88	11.18	0.70	1
Stability test (1:1) (thermocycler)					
24 h	9.97	10.02	10.00	0.02	1
72 h	9.98	10.04	10.05	0.03	1
Absorbance (490 nm)					
Stability test (1:1) (spectrophotometer)					
Day 0	11.99	12.12	12.13	1	1
1 month	26.29	26.08	25.95	2.14	1
6 month	200.84	198.28	188.69	16.20	1

^a^1:1 propylene glycol/glycerin = 50% propylene glycol and 50% glycerin by volume prepared using mass

8:2 propylene glycol/glycerin = 80% propylene glycol and 20% glycerin by volume prepared using mass

2:8 propylene glycol/glycerin = 20% propylene glycol and 80% glycerin by volume prepared using mass

#### Stability tests

For stability tests, three parameters of the e-liquid RM were considered: range of temperature, change in nicotine concentration, and color stability (Fig. 1). The temperature of the e-liquid RM was measured at rest and immediately following 5 min of e-cigarette use in our operating conditions (2.8 Ω heating element at 3.6 V: total of 4.63 W) using a digital thermometer. To assess stability of the e-liquid RM under the temperature change, 1:1 propylene glycol/glycerin e-liquid RM was exposed to 21 C° (at rest temperature) for 90 s and 50 C° (after 5 min of use) for 90 s over 24 and 72 h time periods by using a thermocycler. Three subsamples were collected from the e-liquid RM and saved for nicotine concentration measurement (Table 1). The color change of the e-liquid RM was quantified by measuring absorbance (490 nm) using a spectrophotometer at day 0, 1 month, and 6 months from the initial preparation date (Table 1).

#### Absence of metal impurities in the reference e-liquid

To show purity, the e-liquid RM was analyzed for metal contaminants using Prodigy-Spec ICP-OES (Teledyne Leeman Labs) according to the manufacturer’s protocol. Testing parameters were as follows: Temperature = 34 °C, Torch gas coolant = 19.9 LPM, Aux = 0.31 LPM, Neubulizer = 34.2 PSI, and Pump = 1.4 mM/min. The concentrations of eight metals (cadmium, chromium, cobalt, copper, lead, manganese, nickel, and palladium) were measured in all e-liquid RM samples.

### Nicotine concentration

Nicotine concentration was measured by the previously established protocol using a HPLC-UV (Agilent, Zorbax column) [34]. Measurement parameters were as follows: Injection volume = 100 uL, Temperature = 25 °C, Flow rate = 1 mL/min, Wavelength = 262 nm, Retention = 4.3–4.5 min. A standard calibration curve was calculated using 0, 3, 10 and 30 μg/mL nicotine.

### E-cigarette testing device

The main testing unit was designed and built by researchers at the ADA Foundation Volpe Research Center (Gaithersburg, MD), in collaboration with researchers at the University of Maryland (College Park, MD) using a custom acrylonitrile butadiene styrene (ABS) enclosure (6”×4”×2”) with a 510 adaptor, a precision wattage meter and power analyzer (Guanglian Town ‘G.T.’ power), and an independent 9 V DC power source. Each component of the main unit was carefully selected from lead-free materials including wires and solder. The main unit has two settings: (1) the e-cigarette user mode and (2) the research mode. In the e-cigarette user mode, power is delivered by a 3.6 V lithium-ion battery similar to how e-cigarette users would use the device as described in e-cigarette online forums. In the research mode, filtered power is delivered by a DC power supply (Model D-612 T, Electro Products). The operator is able to increase or decrease the voltage delivered to the main unit in 0.2 V increments. Real-time voltage (V), current (A), and power (W) are displayed on a LED screen on the main unit. We used a 2.0 ml Vivi Nova tank system with a heating element for the proof-of-principle experiments described here. Based on the resistance of the heating element, we used a conservative power setting of 3.6 V (total of 4.63 W based on *P* = V^2^ x R) determined by online “vaping power charts” for all of our experiments. Prior to running the e-cigarette, we made sure that the wick was fully saturated with e-liquid by allowing ≥15 min incubation period after each fill.

### E-cigarette aerosol generation

We used two different methods to capture e-cigarette aerosol depending on specific downstream applications: (1) chemical analysis by Gas Chromatography Mass Spectrometry (GC-MS), and (2) detection of metal(s) by Inductively Coupled Plasma - Optical Emission Spectrometry (ICP-OES).

To capture e-cigarette aerosol for GC-MS analysis, we extracted aerosol directly by connecting a 50 mL glass syringe (Micro-mate) to the e-cigarette mouthpiece. 100 mL e-cigarette aerosol was immediately injected into a 22 mL GC headspace vial with Polytetrafluoroethylene (PTFE)/Silicone rubber septa (Perkin Elmer) using an 18 gauge needle. We used 1 cm^3^ GC glass wool (Sigma Aldrich 20384) as a filter between the mouthpiece and the syringe tip to prevent inadvertent e-liquid aspiration into the syringe space which can lead to clogged GC inlets.

To determine presence of metal(s), we collected the e-cigarette aerosol (15 puffs or 150 puffs) in 30 mL of deionized water (minimum volume allowed) using a gas condenser (Pyrex 1760–125). To prepare for ICP-OES analyses, we prepared 2% HNO_3_ samples by adding 0.35 g of 69% HNO_3_ to 11.65 g of the deionized water containing e-cigarette aerosol. The presence of eight metals that are commonly found in heating elements was evaluated: cadmium (Cd), chromium (Cr), cobalt (Co), copper (Cu), lead (Pb), manganese (Mn), nickel (Ni), and palladium (Pd). The final results are shown in concentration (mg/L) after considering the dilution factor of the collecting liquid medium.

In this study, all tests were performed with the published physiological human e-cigarette puffing topography: 50 mL puff volume in 4 s puff duration every 18 s [35]. Unless otherwise mentioned, all experiments were performed at a constant laboratory temperature (21 °C).

### Chemical characterization of e-cigarette aerosol

All chemical measurements were performed using PerkinElmer Clarus 680 GC with MS Detection (PerkinElmer, Waltham, MA) fitted with a Velocity DB 5 column (PerkinElmer N9306325). Testing parameters of the GC method were as follows: Sampling method = manual headspace, Inlet temperature = 210 °C, Carrier gas = 1.43 L/min, Split = 1:5, Temperature ramp = initial: 40 °C, hold 3 min, 6 °C/min to 300 °C, hold for 3 min, and Total analysis time = 49.33 min. Testing parameters for the MS method were as follows: MS detector = PerkinElmer Clarus, ionization source = El, Polarity = positive, Mass range = (44 to 600) m/z, Acquisition type = centroid, Solvent delay = (0.00 to 2.00) min, and Analysis time = (2.00 to 49.30) min.

### Physical characterization of e-cigarette aerosol

All physical measurements were performed using a TSI Aerodynamic Particle Sizer (APS 3321) according to the manufacturer’s protocol. Aerosol generated by the e-cigarette testing device was released into a 4 L glass smoking chamber by negative pressure generated by a vacuum source at 1.0 L/min. The APS sampling probe was connected directly to the smoking chamber, and was programmed to run three consecutive samples for 20 s with a 1 min break between each cycle.

### Statistical analysis

Concentration measurement, absorbance, and metal detection were quantified using mean ± standard deviation (S.D.) from three independent measurements. Each experiment was repeated in triplicate by calibrated operators. All statistical analyses were conducted using the MaxStat 3.6 statistical software (Jever-OT Cleverns, Germany). The significant level was established as *p* < 0.05.

## Results

### E-liquid Reference Material

#### Gravimetric preparation

Gravimetric preparation of e-liquids provides for exceedingly precise compositional concentrations and ratios [36]. By implementing the Standard Operating Procedure described in Methods and Fig. 1, we demonstrated that highly accurate e-liquid RM can be formulated (a standard deviation of 0.15 mg or less as shown in Table 1).

#### Homogeneity tests

To test uniformity of e-liquids, we examined homogeneity of the reference e-liquid by (i) measuring the time required to reach complete homogeneity and (ii) characterizing secondary separation properties and miscibility of e-liquids. We defined that the e-liquid has reached complete homogeneity when nicotine concentrations of the subsamples were statistically equal. For 1:1 and 8:2 propylene glycol/glycerin e-liquid RMs, we found that by 5 min, the nicotine concentrations were not significantly different (*t*-test, *p* > 0.05). However, propylene glycol and glycerin still existed as two-phase at 20 min. After 1 h rotation, the e-liquid was in complete homogeneity which was confirmed by the nicotine concentration and having a single liquid phase (Table 1). However, homogeneity could not be achieved for 2:8 propylene glycol/glycerin e-liquid RM even after 6 h mixing based on the standard deviation fluctuation (S.D. = 0.70 after 6 h). Secondary separation measurements for 24 h and 2 weeks showed that composition uniformity of the e-liquid stayed intact up to 2 weeks (Table 1).

#### Stability tests

E-liquids are subjected to a wide range of temperature by e-cigarette devices. We measured the temperature of the e-liquid RM sample at rest and after 5 min of use and found that the e-liquid in the tank was subjected to temperatures between (21 to 50 ± 3) C° in our operating conditions (2.8 Ω heating element at 3.6 V: total of 4.63 W). Under the thermocycling conditions (21 C° for 90 s and 50 C° for 90 s over 24 h and 72 h time periods), we found that the subsamples remained homogenous up to 72 h without secondary separation compared to a control.

At day 7, the color of the e-liquid began to change from clear to yellow. The change progressed linearly with time. The absorbance (490 nm) of the e-liquid RM increased by 2- and 16- fold by 1 month, and 6 months respectively compared to a control (Table 1).

#### Detection of metals in the reference e-liquid

High quality RM should not contain other contaminants. To verify that the e-liquid RM is free of metal contaminants, the levels of eight metals were quantified. We found that the levels of eight metals were below the limit of detection (1 ppb by U.S. Environmental Protection Agency Method 200.8) in all e-liquid RM samples.

#### Applications

For best results, we provide the following information regarding the appropriate use of our e-liquid Reference Material:

- Prepare the reference e-liquid immediately prior to use and store up to 1 week in room temperature, away from light and moisture.
- Use a glass round bottom vessel to homogenize. A vessel with acute ends (e.g. centrifuge tubes) traps glycerin and requires longer time to mix.
- Use minimum 6:1 vessel to liquid ratio to provide enough surface area for liquids to be mixed.
- Set a vertical rotator at ≤ 5 r.p.m. Faster speed leads to inadequate mixing.
- RM can be customized to meet individual needs of users as long as the guiding principles described in Fig. 1 are followed. For example, some users may desire other additives such as diluents and/or flavorings in their RM. In such a case, homogeneity and stability parameters should be reestablished.
- More detailed information on development and practical usage of RMs and theories behind homogeneity and stability tests can be found in ISO Guide 35:2006(E) under chapter 6: “Evaluation measurement uncertainty”, chapter 7: “Homogeneity study”, chapter 8: “Stability study”; British Standards Institution (BSI) PAS 54115:2015 under chapter 4: “E-liquids”; and Association Française de Normalisation (AFNOR) XP D 90-300-2 under chapter 4: “General requirements and test methods related to e-liquids”.
- Troubleshooting: typical sources of uncertainty associated with RMs are instrument effects (e.g. calibration of scale and pipettes), reagent purity, measurement conditions (e.g. temperature and humidity), operator effects (e.g. training) and random effects. When there is an observed measurement error that cannot be easily explained, one should refer to the ISO Guide to the Expression of Uncertainty in Measurement (GUM) for more information.

**Table:** 

**E-liquid RM Guidelines At-A-Glance Quick Reference**	
**Step 1**	Start with high quality chemicals
**Step 2**	Use gravimetric preparation method
**Step 3**	Consider the following tips:
	• Use a glass round bottom vessel with 6:1 vessel to liquid vol. ratio • Homogenize ≥ 1 h using a vertical rotator at ≤ 5 r.p.m.
**Step 4**	Prepare fresh and use it within 1 week
**Step 5**	Trouble shooting references:
	• ISO Guide 35:2006(E) • BSI PAS 54115:2015 • AFNOR XP D 90-300-2 • ISO GUM

### Characterization of aerosol generated from the reference e-liquid

#### Chemical by-products in the aerosol

When 50 mL aerosol generated from the reference e-liquid was analyzed using GC-MS, 16 chemical by-products (Table 2) were identified in the e-cigarette aerosol. According to the Globally Harmonized System of Classification and Labeling of Chemicals (GHS), seven chemicals were classified as “dangerous chemicals,” and five carried “warning” labels. Material Safety Data Sheets (MSDS) of the chemicals showed eight may cause acute toxic effects when ingested, and eight may cause respiratory irritation at high concentration (Table 2). Chemical by-products were ranked by Signal-to-Noise (S/N) ratios based on Total Ion Chromatogram (TIC) intensity peak values.

**Table 2:** Chemical by-products found in reference aerosol

Name	GHS signal word	Hazard classification	CAS number	S/N ratio based on TIC peak intensity
Nicotine	Danger	Acute Toxicity 2 Acute Aquatic Toxicity 1 Chronic Aquatic Toxicity 1 Fatal if swallowed or in contact with skin	54–11–5	115578.89
Nicotyrine	Danger	Skin Irritation 3 Acute Toxicity 3 Respiratory Irritation 3	487–19–4	1405.67
Propylene glycol	None	None	57–55–6	709.6
4–Pyridinecarboxaldehyde	Warning	Skin Irritation 2 Eye Irritation 2A STOT SE 3 Combustible liquid Harmful if swallowed May cause respiratory irritation	872–85–5	74.1
2,4,7-trimethyl-1,8-naphthyridine	NF	NF	14757–44–9	59.46
1-Butanol	Danger	Flammable liquid 3 Acute Toxicity 4 Skin Irritation 2 Eye Damage 1 STOT SE 3 Harmful if swallowed May cause respiratory irritation May cause drowsiness	71–36–3	31.09
Cotinine	Warning	Acute Toxicity 4 Skin Irritation 2 Eye Irritation 2A STOT SE 3 Harmful if swallowed May cause respiratory irritation	486–56–6	27.66
5-Methyl-2-heptanol	Danger	Acute Toxicity. 3 Harmful if swallowed	54630–50–1	25.07
1-Methoxy butane	Danger	Flammable liquid	628–28–4	24.8
3-(3,4-dihydro-2H-pyrrol-5-yl) pyridine	Warning	Acute Toxicity 4 Skin Irritation 2 Eye Irritation 2A STOT SE 3 Harmful if swallowed May cause respiratory irritation	532–12–7	22.44
1-(4-pyridinylmethyl)-1H-pyrazol-5-amine	Danger	Acute Toxicity. 3 Harmful if swallowed	3524–31–0	18.12
2,3-dihydro-1H-inden-1-one	None	None	83-33-0	17.01
2-methyl 2-pentanol	Warning	Flammable liquid 3 Skin Irritation 2 Eye Irritation 2A STOT SE 3 May cause respiratory irritation	590–36–3	14.99
3-Ethyl-5-hexen-3-ol	Danger	Skin Irritation 2 Eye Damage 1 STOT SE 3 Acute Aquatic Toxicity 3 Chronic Aquatic Toxicity 3 May cause respiratory irritation	1907–46–6	8.79
6-Methyl-1,2,3,4-tetrahydroquinoline	Warning	Acute Toxicity 4 Skin Irritation. 2 Eye Irritation 2A STOT SE 3 Harmful if swallowed May cause respiratory irritation	91–61–2	8.79
1-(p-Toluidio)-1-deoxy-beta-d-idopyranose	NF	NF	2870–82–8	8.75

S/N ratio = Signal-to-Noise ratio

TIC = Total Ion Chromatogram

STOT SE = Specific Target Organ Toxicity, Single Exposure

NF = Not found

#### Physical properties of the reference aerosol

**Fig. 2:**
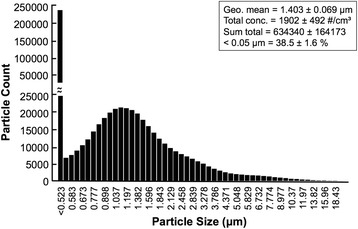
Particle distribution of reference aerosol (20 s sampling schedule, 1.0 L/min sample flow rate)

Aerodynamic particle size and distribution analysis showed that the raw particle count of the reference aerosol was 634,340 ± 164,173 particles/sample (in 0.333 L). The number of particles below 0.5 μm in diameter (within Fine Particulate Matter range defined by the 2012 National Ambient Air Quality Standards for Particle Pollution, U.S. Environmental Protection Agency) was (244,020 ± 61,009) which accounts for 38% of total aerosol. The geometric mean and total concentration were (1.40 ± 0.06 μm) and (1903 ± 492 particle/cm^3^), respectively. The overall distribution is shown in Fig. 2.

#### Presence of metals in the aerosol

**Fig. 3:**
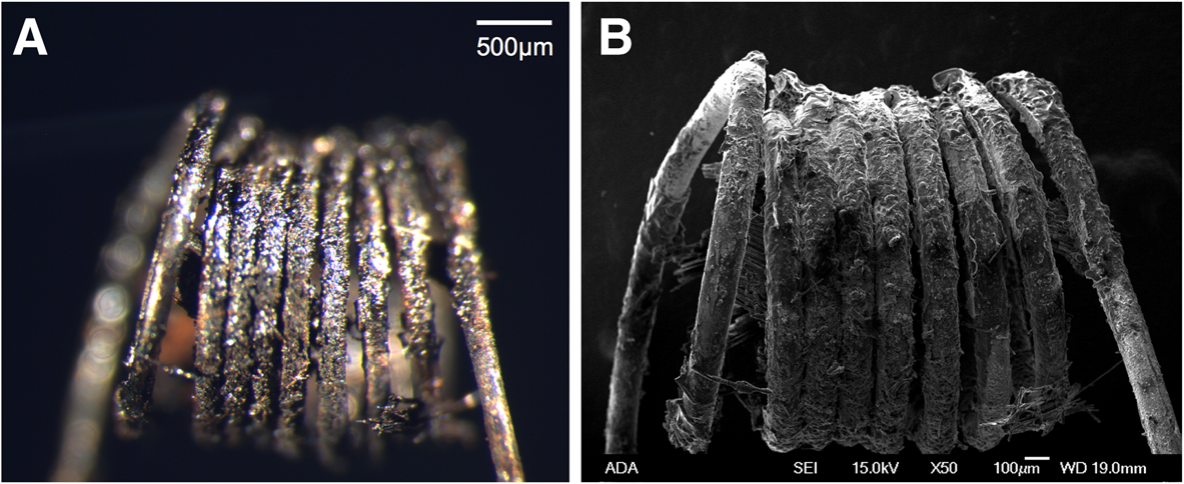
A heating element after 20 h of use **a** the image was taken by using a stereoptical light microscope (bar = 500 μm) and, **b** by a scanning electron microscope (bar = 100 μm)

The reference aerosol was analyzed for the presence of metals in 750 mL (15 puffs) and 7.5 L (150 puffs) aerosol using Prodigy-Spec ICP-OES. The levels of the aforementioned eight metals in 750 mL (15 puffs) and 7.5 L (150 puffs) aerosol samples were below the limit of ICP-OES detection when a new e-cigarette tank was used. Approximately after 4 months of testing (20 h total usage), the e-cigarette had accumulated a noticeable amount of e-liquid residue on its heating element (Fig. 3). Although the e-cigarette was operating normally without obvious signs of a mechanical failure (e.g. reduction of aerosol production, e-liquid leaking or flooding, or gurgling noise), we were now able to detect lead (0.097 ± 0.003 mg/L) and manganese (0.001 ± 0.000 mg/L) from 7.5 L (150 puffs) aerosol.

## Discussion

We developed an e-liquid Reference Material and characterized its physicochemical properties to enhance the reproducibility and promote independent verification in e-cigarette research.

Our results show that a highly pure and accurate e-liquid standard can be formulated when a proper Standard Operating Procedure is implemented (Fig. 1). Our homogeneity and stability tests revealed that the e-liquid RM is stable in room temperature up to 1 week, homogeneous up to 2 weeks and stable up to 3 days in our defined operating condition ((21 to 50) C°, 2.8 Ω heating element and 3.6 V tank system). However during the e-liquid preparation process, propylene glycol and glycerin are likely to adhere to pipette tips, mixers, and containers due to their moderate and high viscosity (0.042 Pa.s and 1.412 Pa.s, respectively) properties. This viscosity may introduce unintended errors in compositional concentration reports, especially when volumetric measurements are used. The gravimetric preparation described here decreases those errors.

The present study has several limitations. We observed a color change in the reference e-liquid at day 7 which led us to presume that oxidation of nicotine was taking place. Although online e-cigarette forums report some e-cigarette users “age” their e-liquids by a process called “steeping”, nicotine oxidation usually is associated with reduced subjective flavor/taste. However biological effects of oxidized e-liquids, if any, are not known at this point. Future studies should evaluate (i) how the oxidation affects physicochemical properties of e-liquids, (ii) if there are any adverse health effects associated with oxidized e-liquids and aerosol generated from them, (iii) how oxidized e-liquids interact with metal components of e-cigarettes (e.g. heating element), and (iv) how the perception of flavors changes with the oxidation process. Future studies should also examine if storing the e-liquid RM at cooler temperature (e.g. –20 C freezer), away from light and moisture will extend its overall service life.

Caution should be taken when interpreting the chemical by-products and metals found in the aerosol as shown in Table 2. Globally Harmonized System (GHS) signal words and hazard classification are based on high concentration and/or repeated exposures of those chemicals. Quantification of the chemical by-products should be considered prior to definitive hazard assessment of e-cigarette aerosol. It is important to point out that metals detected in the aerosol are originating from the e-cigarette device and not from the reference e-liquid. Currently, there is no objective way of determining a lifespan of e-cigarettes. Although online e-cigarette forums recommend replacing the atomizer at the first sign of a mechanical failure or metal taste, the sensitivity of taste varies among individuals, and the level of metal exposure could already be critical if the user can taste metal in the e-cigarette aerosol. Moreover, types of metals used to make heating elements (e.g. nickel, iron-chromium-aluminum, stainless steel, titanium or nickel-chromium), how devices are used (e.g. exposures to rapid heating and cooling cycles), and how heating elements are cleaned (e.g. dry burning or chemical washing) may accelerate the degradation process of e-cigarette devices and heating elements. Studies should further investigate the level of metals as a function of time and usage of e-cigarettes.

Furthermore, the authors acknowledge that the quality and quantity of chemical by-products in e-cigarette aerosol will depend on the device and conditions used to generate the aerosol. New devices are constantly being introduced to the general public with updated technologies (e.g. pyrex tubes, bottom coils, adjustable airflow, sub-ohm heating elements, etc). It will be very difficult to test all of them within a short time frame which reinforces the need for development of standard testing devices and reference materials.

Despite limitations, this study provides insight into why inconsistencies in material composition and concentration are observed in many commercially available e-liquids. The current e-cigarette research experimental designs could also benefit from having standards such as the e-liquid RM described here. The e-liquid RM can be used to check the quality and metrological traceability of commercial e-liquids during a pre-market testing period, instrument calibration, toxicological risk assessment, and safety and efficacy of e-cigarette devices. Ultimately universal e-cigarette testing methods based on a rigorous consensus process from academia, the industry and regulatory government agencies will contribute to understanding and assessing the health risks of e-cigarettes, protection of public safety and promote dissemination of scientifically relevant information in a timely manner.

## Conclusions

A high quality e-liquid RM was formulated by adopting the ISO Guide 35:2006E. Physicochemical properties of the reference e-liquid and its aerosol were determined under conditions closely resembling human modeling of e-cigarette puffing topography. Our results indicate that the reference e-liquid may be used as a part of universal e-cigarette testing methods to enhance chemical, physical, and biological evaluations of e-liquids and e-cigarette devices. Our efforts are consistent with the recent U.S. Food and Drug Administration (FDA) “Deeming Tobacco Products Amendment” (Docket No. FDA-2014-N-0189) and European Union Tobacco Products Directive (2014/40/EU) which in unity calls for better e-cigarette regulations, including transparency in manufacturing and increasing the quality of e-liquids and the safety of e-cigarette devices. The use of universal e-cigarette testing methods and its strategic implementation warrant further study.

## Abbreviations

ABS: Acrylonitrile butadiene styrene
ACS: American chemical society
ADA: American dental association
APS: Aerodynamic particle sizer
BSI: British standards institution
CDC: U.S. Centers for disease control and prevention
EU: European Union
FDA: U.S. Food and Drug Administration
GC: Gas chromatography
GC-MS: Gas chromatography - mass spectrometry
GHS: Globally harmonized system of classification and labeling of chemicals
GUM: ISO Guide to the expression of uncertainty in measurement
HPHCs: Harmful and potentially harmful constituents
HPLC-UV: High performance liquid chromatography - ultraviolet vis absorbance detector
ICP-OES: Inductively coupled plasma - optical emission spectrometry
ISO: International standards organization
MS: Mass spectrometry
MSDS: Material safety data sheet
NIST: U.S. National institute of standards and technology
PPB: Parts per billion
PTFE: Polytetrafluoroethylene
RM: Reference material
RPM: Revolutions per minute
S/N: Signal-to-Noise ratio
SD: Standard deviation
SOP: Standard operating procedure
TIC: Total ion chromatogram
